# Supplementary material for: Mouse lung automated segmentation tool for quantifying lung tumors after micro-computed tomography
Source: PLoS One. 2021 Jun 17;16(6):e0252950. doi: 10.1371/journal.pone.0252950 (PMC8211241; doi:10.1371/journal.pone.0252950)
Supplement: Attachment — Submitted filename: Response to Reviewers - PONE-D-20-38790-R1.docx [file pone.0252950.s007.docx]

**Author’s Response to Reviewer's Questions (highlighted in RED)**

**Reviewer #1:**

We thank the reviewer for constructive comments and feedback on the manuscript draft. We have made every effort to address your concerns by providing additional details, figure/s, reference/s. We hope that the revised draft is acceptable to the reviewer. Below is our point-by-point response to the questions/concerns raised by the reviewer #1.

In this paper, the authors describe a new method that enables quick automatic segmentation of lung tumor in small animal models that might have use in drug studies. Experiments seem to have been conducted properly and results are interesting. However, the statements done regarding the results obtained are over rated and more self-critic is recommended.

My comments follow:

(1) Line 42: Please define what the intermediate region is. There is no definition of it in the text and it must be given in detail.

**Response:** An expanded description of the intermediate and other regions is described in the methods section of the revised draft (Line 160-167, draft with track changes).

(2) Lines 107 and 109: Attention to the subscripts for carbon dioxide.

**Response:** We have corrected in the revised manuscript draft.

(3) Line 134: is it resolution or pixel size? The images suggest it is pixel size, not resolution.

**Response:** The text is correct, it is ‘resolution’.

(4) Line 105: it is Feldkamp, not Fledkamp.

**Response:** We have corrected in the revised manuscript draft.

(5) Lines 144-147: what is the size of a “small tumor nodule”? Precise values for “small”, “medium”, and “large” are needed here. An explanation is given in the legend of Fig 1 and more should be written in the main manuscript text.

**Response:** We thank the reviewer for this good question. The manual qualitative scores are ordinal bins based on visual assessment to ensure a spectrum of tumors and total tumor burden. Thus, we have not measured size while scoring the tumor burden. This evaluation is analogous to clinical radiological assessments. Although such evaluation provided us quicker assessment, was not quantitative to inform moderate to subtle differences in tumor volume after treatment. The MLAST and manual segmentation are more quantitative and more able to discern subtle volumetric changes than the ordinal binning, with MLAST having an added advantage of being efficient and non-subjective.

(6) Line 128 and 279: KL-GEM or KL GEM?

**Response:** We have corrected in the revised manuscript draft.

(7) Line 148: write Amira 6.3 (ThermoFischer...).

**Response:** We have corrected this in the revised manuscript draft and incorporated the vendor info in line 142.

(8) Line 160 - 162: please give a precise definition of a “significant decrease in the percentage of lung volume” must be given. As it is written, it is too imprecise.

**Response:** This section has been updated to address the concern by the reviewer.

(9) Line 205: Use “h”, not “hours”.

**Response:** We have corrected in the revised manuscript.

(10) Line 230: Title seems to be incomplete. It is a comparison of what to what?

**Response:** We have modified the title in the revised manuscript.

**(11)** Figure 4 is hard to understand. Fig 4A: it is said it is a comparison of manual score with MLAST, but only the results of manual segmentation are shown (or only MLAST? The figure and the legend apparently do not match).

**Response:** We have amended the figure and the figure legend to give more clarity. Fig 4A shows the relative values of the three tissue types segmented by MLAST across the manually scored samples. You can appreciate that samples with greater manual score had higher soft tissue and intermediate values, whereas the lung tissue decreased with higher manual scores. The results section provides a comprehensive explanation on this data.

Fig 4B: Why heart + tumor volume manually estimated should have a correlation with MLAST-based estimation of soft tissue + intermediate? Maybe this is obvious for the authors, but is not written in the text.

**Response:** We have amended the figure, figure legend and results section to give more clarity. Additionally, MLAST segmented tissues is explained in the Methods section “Automated Analysis using MLAST”.

Again, the same for Fig. 5. A and B. A comparison of manual segmentation to MLAST segmentation is the core of the paper, tough no explanation is given for why this comparison (heart + tumor volume vs. soft tissue + intermediate) makes sense scientifically and is the best that can be achieved in the context of this work.

**Response:** Hope the above explanation for Fig 4A/B provide better context to the reviewer. In Fig 5A, we have added another mCT imaging procedure with administration of contrast agent. Here manual segmentation after contrast agent administration is compared with MLAST and manual segmentation without contrast agent. We have made amendments in the results section to provide more clarity.

Then in Fig 5 C, the comparison of MLAST and histology only considers the tumor area for histology and soft tissue + intermediate for MLAST. Why is there a different comparison for histology?

**Response:** Histology is one of the most common methods used in lung tumor evaluation, especially labs that do not have mCT imaging capability. Thus, we thought it is important to compare, correlate and show case the merit of MLAST and tumor area by traditional histology.

(12) Line 250: I believe there’s something wrong with this sentence.

We have made changes to address the reviewer’s concern.

(13) Line 261: correct “side-by-side”.

**Response:** Corrected in the revised draft

(14) Line 279: a definition of KL-GEM model is needed in the methods part.

**Response:** The definition can be found in the animal models section of methods.

**(15)** Line 287: “Figure 5B shows the segmentation of lung. The results of manual and MLAST segmentation are obviously different and this different has to be addressed in the text. Of course, manual and MLAST segmentation are going to be different, but are the differences relevant? If not, why? The authors are interested in the statistical differences between the groups studied, but these differences in segmentation cannot be overlooked and the minimum number of scanned animals to give relevant results should be given.

**Response:** We agree with the reviewer’s comments. The considerable y-axis offset shows that this difference is consistent. The difference is primarily due to native, normal soft tissue structures (vascular and esophagus), which is difficult to manually segment in a reproducible fashion. This point is well explored in lines 363-365 of the discussion. In the revised draft, we also added and re arranged the text in results section to provide clarity.

(16) Line 296 and 303 (and other parts in the text): micro-CT or mCT? Choose one or other, because using both terms shows lack of consistency.

**Response:** Have adopted to the term “mCT” and have amended the whole manuscript.

(17) Line 302: what is vehicle-treated group? Is it the control group? Clear information in the methods part is needed and the use of the term “control group” is recommended.

**Response:** The term “control” has been added for clarity.

(18) Line 318: What is “finer granularity to scan evaluation”? A clear definition must be given.

**Response:** Text has been updated with the phrase “improved quantification” for clarity.

**(19)** Line 320: a comparison of the images in Fig 3 (segmented-top and original-bottom) shows that MLAST overestimates the boarder of the bones. From the images, I cannot say for sure if the same happens with boarders within soft-tissue, but a rough visual analysis suggests unclear boarders between tissue types. Thus, I definitely do not agree with “This effect is potentially useful at lesion margins where the analyst is unable to distinguish between proliferating tumor edges and other lesions such as tumor compression induced atelectasis [22], peritumor edema associated with pseudoprogression [23], or at tumor-organ interfaces (as shown in Figure 5B).” More explanation and less speculation are needed here. Also, maybe the authors should consider presenting **Fig 3 as Fig 5 B (**superposition of the segmented areas in transparent colors on the micro-CT slice).

**Response:** We thank the reviewer for the suggestions. We have amended Fig 3 and have included super-imposed images of MLAST and mCT. We have updated the Figure 3 legend to provide clarity for the reader. The bone mask is generated from multiple slices as described in methods on lines 165-167. As described in the text, this smoothed the spline function and created a better segmentation of the thoracic cavity. This does not apply to the other tissues.

(20) Line 327: I recommend to add more references to the “semi-automated and fully-automated approaches”.

**Response:** We have additional references in the revised draft.

**(21)** Lines 357 and 370: the same statement is given twice, at the beginning and at the end of the paragraph: “Comparison with manual segmentation volumes demonstrated the accuracy of tissue segmentation by MLAST”. Once should be enough. However, as commented before, I see a problem in this comparison and, thus, this statement is questionable. A proper explanation and scientific basis for this comparison must be given.

**Response:** We have removed the repeat sentence as suggested by the reviewer. We have amended the results section and figure legend to provide further explanation (as explained for your point #15).

(22) Line 373: “compared”, not “correlated”.

Changed.

**(23)** Line 357-358: I do not agree with “Comparison with manual segmentation volumes demonstrated the accuracy of tissue segmentation by MLAST.” Fig 5 C shows some statistical correlation in the measurements, but does not really show accuracy. Is it possible to show figures such as Fig. 4 in [https://doi.org/10.1038/s41598-018-37394-w?](https://urldefense.proofpoint.com/v2/url?u=https-3A__doi.org_10.1038_s41598-2D018-2D37394-2Dw-3F&d=DwQGaQ&c=UE1eNsedaKncO0Yl_u8bfw&r=TUvk4PA32fah7abuWf0tRR9RyKsi6SQQIJNg28fyL_g&m=4dQjg4FWteIlyg5Xv0Ql_cIXypHHj5dsKW6ahfpURbU&s=UpfG_RtSNEI-u3RloJtt_0aDs3aK41z9J5gMPXAfk0s&e=) This would show accuracy. More self-critic is recommended.

**Response:** This point by the reviewer is well taken. Unfortunately, since our mCT imaging is in vivo we cannot perform the comparison as done in the manuscript by Bidola P et al., We have updated the text as the high correlation with a y-axis offset is better described as ‘precision’ rather than ‘accuracy’.

(24) Line 382 - 384: Why is MLAST a “dynamic measurement”? Are the “subtle differences within scoring categories” relevant or they are an error of the method? How to tell the difference?

**Response:** The offending phrase has been removed in the revised draft. Yes, the subtle differences important while evaluating efficacy, dose-response relationships. We really need a very strong difference or therapeutic effect to see statistical difference in the ordinal scoring system and MLAST offers ability to identify subtle differences. Albeit not presented here, we do have data from many portfolio studies that has helped us make this inference.

(25) Line 389-392: I agree that MLAST “has much potential value to the process of drug development”. It for sure can increase provide quick results that, within different populations, has a statistical relevance. However, I do not agree that the results indicate that it is more sensitive than the other methods used.

**Response:** We’ll clarify that we are comparing MLAST to the ordinal scoring methods. We have amended the text to reflect this correction.

(26) Legends of Fig. 4 and 5: The use of the word “validation” is questionable. The figures show a comparison of the results, but do not show a proof, or a confirmation, as the word “validation” suggests. Use of “comparison” is more appropriate.

The wording “validation” is now changed to “comparison”

**Reviewer #2:**

We really thank the reviewer #2 for very positive feedback. We are very glad that you found our manuscript well written, interesting, and will make a difference in the field. Please see our response to your minor comments. Hope this will be satisfactory to you.

The authors describe a workflow to automatically analyze the lungs of mice that were imaged using µCT. This work describes a practical solution to a task in pharmaceutical research that should inspire the community to use processes that accelerate the search of new treatments against cancer. Tumor growth will change the lung tissue composition and will become visible in the x-ray images. Based on the setup described here, tumor growth should give a strong signal and this would allow different ways for an analysis such as visual classification, or a densitometric analysis (i.e. shifts in voxel intensity histograms), or a size measurement after lung segmentation. The authors use a size measurement after lung segmentation and describe the steps needed for a robust and fully automized procedure.

The results are compared to an analysis based on ground truth segmentation generated by human experts, a tumor burden score also performed by a human expert, and readouts generated from histology. The correlation proves the usability of the authors approach.

In Fig 2A a histogram of density values is shown. Histogram analysis seems to be a common tool in x-ray image analysis and would probably give information about the health state of lungs. The authors should discuss why they did not use the densitometry data to also analyze tumor presence or have they tried this with unsatisfactory results.

**Response:** We are sorry if our explanation in methods section resulted in any confusion. As mentioned, the segmentation of the tissues is based on its density. The graph in Fig2A shows the three tissues in the lung space (healthy lung – Blue; Soft tissue – Green and Intermediate – Cyan). This was applied to the tomographic data.

Fig 5A shows the weak part of the MLAST approach. Small tumor nodules in lung lobes that can easily be seen and used in a scoring scheme, can also be marked in a manual segmentation, but will have little effect on volumetric measurements. The smoothing effect of the clustering in MLAST leads to a significant offset. In the discussion the authors explain in detail all the effects caused by the different analysis methods used. However neuronal networks are not used for this work. The authors argue that a deep learning approach to classify or segment the lung was not feasible because of missing ground truth in the public domain and therefore not considered. For the evaluation of their MLAST procedure they generated over 3500 ground truth images for segmentation and used 446 lungs for scoring, a source for ten thousands of images for a classification training. In the case of tumor burden in lungs this is sufficient material for an application using deep learning. The authors should rethink their arguments.
This manuscript describes in great detail a practical solution to a segmentation task. Alternative solutions that might even be easier and faster, are not discussed, but should be worth a few sentences.

**Response:** Thank you for pushing to further explore deep learning approaches in the text. This is an area that we will explore in the near-future. We’ve added a new paragraph to reflect your comments in the discussion section.
